# Mediation of the risk of serious police or legal trouble in adults with a mental disorder

**DOI:** 10.1017/S003329172610511X

**Published:** 2026-07-30

**Authors:** Alec Buchanan, Elina Stefanovics, Ran Wu, Thomas J. McMahon, Ralitza Gueorguieva

**Affiliations:** 1 Law and Psychiatry Division, Department of Psychiatry, Yale University School of Medicine, New Haven, USA; 2 Division of Addictions, Department of Psychiatry, Yale University School of Medicine, New Haven, USA; 3 Department of Psychiatry, Yale University School of Medicine, New Haven, USA; 4 Child Study Center, Yale University School of Medicine, New Haven, USA; 5 Department of Biostatistics, Yale University School of Public Health, New Haven, USA

**Keywords:** behavior problems, diagnosis, epidemiology, psychosis, legal trouble, violence, mood disorder

## Abstract

**Background:**

Adults with mental disorders more frequently report getting into serious trouble with the police and the law than do adults with no mental health diagnosis. The mechanisms underlying this statistical association remain unclear.

**Methods:**

The study used data from a US population survey to examine whether the previously recognized association between psychiatric diagnosis and getting into serious police or legal trouble is mediated by impaired psychosocial function or psychiatric symptoms. The predictor variable was psychiatric diagnosis. The mediator variables were two forms of psychosocial function, social and role-emotional, and the presence of recent symptoms. The outcome variable was police or legal trouble.

**Results:**

When known risk factors are included as covariates, the association between mental disorder and serious police or legal trouble over the past year is maintained and is significantly mediated (*p* < 0.05) both by social and role-emotional function (social, excess relative risk [ERR] 1.42 [95% CI: 0.96, 1.88]; role-emotional, ERR 1.45 [95% CI: 0.98, 1.91]) and by recent symptoms(ERR 1.63 [95% CI: 1.13, 2.13]). The contribution of mediation varies by diagnosis.

**Conclusions:**

Previous research suggested that it is unusual for psychiatric symptoms to lead directly to violence. These results suggest that symptoms and signs of mental disorder nevertheless contribute indirectly to violence and other adverse outcomes. The identification of these mediators may, in turn, suggest new approaches to reducing the risk of those outcomes. Future research should further examine and describe the mechanisms that underlie these and other forms of mediation.

## Introduction

Being diagnosed with a mental disorder is associated with a significantly increased risk of a range of forms of police and legal trouble, including arrest and imprisonment. US population survey data suggest that people with mental illness are five times as likely as people with no mental disorder to have been in serious police or legal trouble over the previous 12 months (Buchanan, Moore, Pittman, & McKee, 2021).

The size of this effect varies according to diagnosis. The effect is greater when mental illness is accompanied by substance use or personality disorder, where community surveys report odds ratios of over 10 compared with the general population (Grann, Danesh, & Fazel, 2008; Van Dorn, Volavka, & Johnson, 2012). Other replicated correlates of police and legal trouble in people with mental disorders include younger age, male sex, and a history of arrest or violence (Fazel et al., 2010; Fazel & Grann, 2006; Swanson, Holzer, Ganju, & Jono, 1990; Swanson et al., 2025; Whiting, Lichtenstein, & Fazel, 2021).

Awareness of these statistical correlations, however, does not on its own make clinical and risk management interventions more effective. One additional type of information that would assist clinicians relates to temporal effects. People’s symptoms do not remain constant but are subject to ebbs and flows. To date, attempts to demonstrate associations between violence and the presence of individual symptoms or constellations of symptoms have generally been unsuccessful (Appelbaum, Robbins, & Monahan, 2000; Buchanan et al., 2013; Skeem et al., 2006). One understudied question with implications for clinical management is whether people with mental disorders are more likely to get into trouble with the law during periods of overall clinical deterioration.

A second, related, type of information that would assist clinicians relates to the mechanisms that underlie the correlations described above. Those mechanisms seem unlikely to be the same for different mental disorders, for instance, for substance use versus other disorders, and perhaps also for psychotic versus nonpsychotic disorders. Even within an individual disorder, it is not known whether police and legal trouble is more strongly associated, for instance, with the recurrence of symptoms or with a deterioration in psychosocial function. Many of the well-recognized correlates of legal trouble, including younger age and past history of antisocial behavior, are not amenable to change. An improved knowledge of the mechanism would help clinicians to target dynamic risk factors.

We investigated whether the known link between lifetime mental disorder and serious legal involvement, including but not limited to violence, is mediated by the presence, at the time of that legal involvement, of (a) impaired psychosocial function or (b) psychiatric symptomatology reaching the threshold for a DSM-5 diagnosis.

## Methods

### Sample and procedure

The National Epidemiologic Survey on Alcohol and Related Conditions (NESARC)-III is a cross-sectional representative survey of the civilian US population sponsored by the National Institute on Alcohol Abuse and Alcoholism (NIAAA). Data were supplied by the National Institutes of Health (https://www.niaaa.nih.gov/research/data-archive-resources/niaaa-controlled-datasets/nesarc-iii-study) at the request of the authors.

NESARC-III conducted in-person interviews with noninstitutionalized US adults, including the residents of group and rest homes but excluding people in prison, between April of 2012 and June of 2013. Interviews were conducted using the Alcohol Use Disorder and Associated Disabilities Interview Schedule (AUDADIS)-5, an extensively tested instrument with good psychometric qualities (Grant et al., 2015). Data were weighted to adjust for nonresponse and were nationally representative with regard to age, sex, and ethnicity. The final sample size was 36,309. Consent procedures were approved by the National Institutes of Health. Detailed descriptions of the methodology have been published (Grant et al., 2015, 2016; Grant, Chu, Sigman, 2014).

### Variables

We conducted a mediation analysis. We follow a convention of mediation analysis to refer to variables here as “predictors,” “mediators,” “co-variables,” and “outcome” (see Table 1). The predictor variable in the mediation analyses was psychiatric diagnosis. Categories were generated for three forms of mental disorder (mental illness, substance abuse, and personality disorder) and for subcategories within these using subjects’ responses on the (AUDADIS)-5.

**Table 1.** Description of sample and study variables (*N* = 36,309) **^a^** Table 1. long description.

Predictor			Mediator						Outcome	
Diagnosis			Recent psychosocial function				Recent symptoms		Serious police or legal trouble^b^	
Diagnosis	*n*	% (of *N*)	Social function (mean)^c^	Social function (SD)	Role-emotional function (mean)^d^	Role-emotional function (SD)	subjects	% (of *n*)	subjects	% (of *n*)
No diagnosis	19,295	53.14	52.36	8.95	49.50	10.12	1525	7.90	161	0.83
Any MD (MI, SU, or PD)	17,014	46.86	47.62	12.04	45.67	11.73	10666	62.69	495	2.90
Any MI	11,923	32.84	46.27	12.64	44.40	12.11	8772	73.57	319	2.68
Schizophrenia/Psychosis	842	2.32	42.60	14.48	40.33	13.80	570	67.7	40	4.75
Any mood disorder	8,293	22.84	45.48	12.90	43.76	12.16	5983	72.15	240	2.89
Major depression	7,095	19.54	46.12	12.56	44.43	11.91	4979	70.18	184	2.59
Dysthymia	2,323	6.40	40.66	14.23	39.21	13.15	1986	85.49	91	3.92
Bipolar 1	698	1.92	41.60	14.17	39.59	12.83	607	86.96	41	5.87
Any anxiety disorder	5,821	16.03	44.90	13.26	43.29	12.45	5045	86.67	175	3.01
Specific phobia	2,269	6.25	45.37	13.10	44.14	12.16	2161	95.24	53	2.34
Social anxiety	1,248	3.44	42.02	13.88	41.40	13.01	1138	91.19	47	3.77
Panic	1,713	4.72	41.58	14.16	40.40	12.84	1454	84.88	60	3.5
Agoraphobia	677	1.86	39.56	14.30	38.18	13.30	637	94.09	25	3.69
Generalized anxiety	2,556	7.04	43.21	13.79	41.66	12.67	2181	85.33	96	3.76
Posttraumatic stress	2,219	6.11	41.52	14.11	40.36	13.15	1981	89.27	94	4.24
Eating disorder	595	1.64	44.49	13.17	43.47	12.33	517	86.89	11	1.85
Any substance use disorder	8,628	23.76	47.89	11.78	46.15	11.45	5336	61.85	350	4.06
Alcohol use disorder	7,785	21.44	47.97	11.71	46.26	11.39	4755	61.08	301	3.87
Drug use disorder	2,855	7.86	45.77	12.63	44.28	12.06	2111	73.94	182	6.38
Any personality disorder	5,746	15.83	43.94	13.35	42.24	12.53	4216	73.37	315	5.48
Borderline	4,301	11.85	42.38	13.61	40.94	12.59	3390	78.82	250	5.81
Antisocial	1,600	4.41	45.53	13.13	43.33	12.73	1136	71	132	8.25
Schizotypal	2,439	6.72	41.96	13.98	40.54	13.13	1929	79.09	148	6.07

*Note*: SD, standard deviation. Psychiatric categories not mutually exclusive. Higher scores indicate better psychosocial function. Shading indicates variables selected for mediation analysis.

a Sample weighted before this analysis to adjust for nonresponse.

b 11 out of 36,309 subjects had missing values.

c 14 out of 36,309 subjects had missing values.

d 16 out of 36,309 subjects had missing values.

The AUDADIS-5 interview includes follow-up questions regarding the timing of symptoms and generates DSM-5 categories for three time periods: “prior to past 12-months,” “past 12-months,” and, when either of these is present, “lifetime” (Grant et al., 2015, 2016). Diagnostic categories are not exclusive, and subjects could be allocated to more than one category except where DSM-5 makes exceptions on clinical grounds. We did not allocate a patient to dysthymia, for instance, if they met criteria for major depression. AUDADIS-5 personality disorder DSM-5 categories were available in the NESARC database for “lifetime” only.

We excluded symptoms that were only present over the past 12-months from the predictor variable because these data were used to generate the mediator variable, recent symptoms. As others have done (Moore et al., 2019), we used the response on the NESARC-III item, “Did a doctor or other health professional tell you that you had schizophrenia or a psychotic illness or episode?,” and the response to a subsequent question as to whether this had happened prior to the past 12-months, to generate a further variable, “schizophrenia/psychosis.”

The mediator variables were psychosocial function (divided into social functioning and role-emotional functioning) and recent symptoms. We measured psychosocial function using the 12-Item Short-Form Health Survey Version 2 (SF-12v2; Gandek et al., 1998). The SF-12v2 generates norm-based disability scores with a mean (SD) of 50 (10) and a range of 0–100. Lower scores indicate greater disability. Social function is defined by the SF-12v2 as the extent to which a subject perceives health problems as having interfered with normal social activities (McHorney, Ware, Lu & Sherbourne, 1994; Ware & Sherbourne, 1992). One item measuring social functioning asks, “During the past 4 weeks, how much of the time have your physical health or emotional problems interfered with your social activities like visiting with friends, relatives, and so forth?”

Role-emotional functioning in the SF-12v2 addresses the extent to which a subject’s symptoms lead them to cut down the amount of time spent on work, to accomplish less than they would like at work, and to carry out daily activities less carefully than usual. Items include, “During the past 4 weeks, tell me how much of the time you have had any of the following problems with your work or other regular daily activities as the result of any emotional problems, such as feeling depressed or anxious,” “How much of the time have you accomplished less than you would like,” and, “How much of the time have you not done work or other activities as carefully as usual?”

Recent symptoms were rated using subjects’ responses on AUDADIS-5 items and on the NESARC-III item, “Did a doctor or other health professional tell you that you had schizophrenia or a psychotic illness or episode,” all relating to the past 12 months. The variable was rated as present when the symptoms were sufficient to meet criteria for any DSM-5 mental illness or substance abuse category.

The covariables were age, sex, and a lifetime history of violence. Age and sex (self-defined) were coded from NESARC-III subject interviews. A lifetime history of violence was measured, as elsewhere (Harford, Chen, Kerridge, & Grant, 2018), using a positive response to any of 7 NESARC-III items relating to violent behavior occurring since the age of 15: stole directly (by mugging or making threats with a weapon); forced sexual activity; got into fights one started; physically hurt someone; exchanged blows in a fight; used a weapon; and hit someone causing injury or hospitalization. The outcome variable, serious police or legal trouble over the past year, was generated using the response to the NESARC-III item, “Please tell me if you have had any of the following experiences in the last 12 months … did you have serious trouble with the police or the law?”

### Statistical analyses

First, we confirmed that the association, noted by others in this and other samples, between mental disorder and legal involvement of various kinds persists when the diagnosis excludes “past year” symptoms and when the outcome is limited to serious trouble with the police or the law over the previous 12 months. We report rates of such involvement for each diagnostic category (Table 1).

Second, we sought to establish whether, after controlling for known risk factors for violence and legal trouble (younger age, male sex, history of violence, and substance use), the remaining association is mediated by impaired psychosocial function or by recent symptoms. To reduce the likelihood of Type 1 error, we limited predictors to a reference group (subjects with no mental disorder), three categories of mental disorders of clinical relevance (any mental disorder, mental illness, and substance abuse), and two diagnoses that are characterized by psychotic symptoms and that have been associated with violence in previous research (schizophrenia/psychosis and bipolar 1 disorder; Fazel & Grann, 2006; Swanson et al., 2025; Whiting et al., 2021).

We performed mediation analyses following the causal inference approach of Vanderweele (Vanderweele, 2014; Valeri & Vanderweele, 2013) and using PROC CAUSALMED in SAS Version 9.4 (SAS Institute, Inc., 2017). The outcome variable in all analyses was serious police or legal trouble in the past 12 months. We fitted separate logistic models for each of the five predictors and with each of the three potential mediations (social functioning, role-emotional functioning, and recent symptoms), also considered separately. We included age, sex, and a history of violence as covariates. The alpha level was set at 0.05.

## Results

Table 1 describes the sample in terms of the predictor, mediator, and outcome variables. It shows that 47% of subjects had a DSM-5 diagnosis of any mental disorder as measured by the AUDADIS-5. Mental illness comprised the largest category, followed by substance abuse and personality disorder. Both types of psychosocial function, social and role-emotional, were significantly reduced in subjects with any mental disorder compared with subjects with no disorder (mean [SD] social = 47.6 [12.0] vs. 52.4 [9.0], *F* [1, 36293] = 1838.8, *p* < .0001; mean [SD] role-emotional = 45.7 [11.7] vs. 49.5 [10.1], *F* [1, 36291] = 1114.8, *p* < .0001). Recent symptoms were significantly more common in subjects with a mental disorder (63%) than in subjects without (8%; *χ* ^2^ = 12168.1, *p* < .0001).

Absent the control for known risk factors that is present in the mediation analyses, subjects with any mental disorder were significantly more likely than those with no disorder to describe having serious trouble with the police or law over the previous 12 months (2.9% vs. 0.83%; *χ* ^2^ = 219.28, *p* < .0001). Similarly, for each of the other categories and subcategories of mental disorder used in the mediation analyses, subjects who met criteria for diagnosis were significantly more likely to describe having serious trouble with the police or law than subjects who did not meet those criteria (any mental illness 2.68% vs. 1.38%, *χ* ^2^ = 75.48, *p* < .0001; schizophrenia/psychosis 4.75% vs. 1.73; *χ* ^2^ = 42.21, *p* < .0001, bipolar 1 disorder 5.87% vs. 1.73%, *χ* ^2^ = 66.32, *p* < .0001, any substance abuse disorder 4.06% vs. 1.11%, *χ* ^2^ = 322.77, *p* < .0001).

Table 2 contains the results of the mediation analyses. Effect sizes are reported as odds ratios (ORs) and total excess relative risk (ERR), each with 95% confidence intervals. For ORs, confidence intervals that do not include 1 correspond to statistically significant effects at the 0.05 significance level. For ERRs, confidence intervals that do not include 0 correspond to a significantly increased risk of the outcome in the corresponding diagnostic group compared to the control group, again at the 0.05 significance level. The total ERR is decomposed in the analysis into natural direct (unmediated) and natural indirect (mediated) effects. Finally, we report percent of the total effect (as measured by ERR) that is due to mediation in each model.

**Table 2.** Mediation analysis^a^ Table 2. long description.

Mediator	Odds ratio (and CI) for 12 m serious police or legal trouble^b^	Excess relative risk (ERR; and CI)	ERR (and CI) due to natural direct effect (NDE)	ERR (and CI) due to natural indirect effect (NIE)	NIE as % of ERR
No diagnosis	Ref				
Social function		Ref	Ref	Ref	
Role-emotional function		Ref	Ref	Ref	
Symptoms 12 months		Ref	Ref	Ref	
Any MD (SU, MI, or PD)	2.48 (2.05, 2.99)				
Social function		1.42 (0.96, 1.88)	1.07 (0.67, 1.48)	0.35 (0.26, 0.44)	24.5
Role-emotional function		1.45 (0.98, 1.91)	1.15 (0.74, 1.57)	0.29 (0.21, 0.37)	20.3
Symptoms 12 months		1.63 (1.13, 2.13)	0.45 (0.13, 0.76)	1.18 (0.88, 1.45)	72.6
Any MI	1.78 (1.51, 2.09)				
Social function		0.70 (0.42, 0.97)	0.40 (0.16, 0.64)	0.30 (0.23, 0.36)	42.7
Role-emotional function		0.71 (0.43, 0.99)	0.46 (0.21, 0.70)	0.25 (0.19, 0.32)	35.7
Symptoms 12 months		1.07 (0.73, 1.42)	−0.01 (−0.20, 0.17)	1.09 (0.85, 1.32)	100
Schizophrenia/Psychosis	2.52 (1.80, 3.52)				
Social function		1.35 (0.54, 2.15)	0.78 (0.16, 1.34)	0.57 (0.35, 0.79)	42.2
Role-emotional function		1.39 (0.57, 2.21)	0.83 (0.20, 1.46)	0.56 (0.35, 0.78)	40.5
Symptoms 12 months		1.66 (0.76, 2.57)	0.84 (0.22, 1.47)	0.82 (0.52, 1.13)	49.4
Bipolar 1	2.01 (1.43, 2.82)				
Social function		1.03 (0.33, 1.72)	0.47 (−0.04, 0.98)	0.55 (0.34, 0.77)	53.8
Role-emotional function		1.01 (0.32, 1.70)	0.48 (−0.03, 0.99)	0.53 (0.32, 0.73)	52.4
Symptoms 12 months		1.32 (0.53, 2.10)	0.38 (−0.09, 0.85)	0.94 (0.60, 1.27)	71.1
Any substance use disorder	2.16 (1.82, 2.55)				
Social function		1.15 (0.79, 1.52)	0.94 (0.61, 1.26)	0.22 (0.16, 0.27)	18.9
Role-emotional function		1.14 (0.78, 1.50)	0.98 (0.65, 1.32)	0.16 (0.12, 0.20)	14.0
Symptoms 12 months		1.19 (0.82, 1.55)	0.55 (0.28, 0.81)	0.64 (0.50, 0.78)	54.0

a *p*-values for odds ratios and excess relative risk all <0.05.

b Incorporating as covariates age, sex and history of violence.

When known risk factors are included as covariates, the odds of serious police or legal trouble remain more than two times higher for subjects with any mental disorder than for subjects without any mental disorder (OR = 2.48, 95% CI: 2.05, 2.99). When social function is included as the mediator, the ERR is 1.42 (95% CI: 0.96, 1.88) and social function mediates 24% of the effect of the mental disorder as a predictor of serious police or legal trouble. When role-emotional function is included as the mediator, the ERR is 1.45 (95% CI: 0.98, 1.91) and role-emotional function mediates 20% of the effect of the mental disorder. With recent symptoms as the mediator, the ERR is 1.63 (CI: 1.13, 2.13) and recent symptoms mediate 73% of the effect of the mental disorder. The contribution of mediation to the ERR varies by diagnostic class, with each of the three mediators accounting for a higher percentage of the ERR in subjects with mental illness than in subjects with a substance abuse disorder.

For each of the five predictors, each of the two measures of psychosocial function contributed less to the ERR (the final column of Table 2 shows that the contribution lay between 14.0% and 53.8%) than recent symptoms contributed (where the contribution lay between 49.4% and 100%). The final column of Table 2 also shows that social and role-emotional functioning account for a greater percentage of the total effect in schizophrenia/psychosis (42.2% and 40.5%, respectively) and bipolar 1 disorder (53.8% and 52.4%, respectively) than it accounts for in mental disorder as a whole (24.5% and 20.3%, respectively).

We constructed cross-tabulations as an aid to interpreting the Results. For subjects with mental illness, 293 out of 319 (92%) who had been in serious trouble with the police or law in the past 12 months described recent symptoms. By comparison, only 8,477 out of 11,602 (73%) of subjects who did not describe such trouble described recent symptoms.

## Discussion

The effect of psychiatric diagnosis on the risk of a subject having serious police or legal trouble in the previous 12 months, after controlling for known risk factors, is significantly mediated both by psychosocial function and, to a greater extent, by psychiatric symptoms. Previous research suggests that it is relatively unusual, outside forensic populations (Penney, Morgan, & Simpson, 2021), for symptoms, such as low mood and anxiety (Peterson et al., 2014), or signs of illness, such as delusions (Appelbaum et al., 2000), to lead directly to violence. These results suggest that illness may nevertheless contribute indirectly to adverse legal outcomes. One mechanism that is consistent with these results is that symptoms and signs of illness damage family and social networks that would otherwise be protective.

The mediation effects of both psychosocial function and symptoms are more pronounced for mental illness than for substance abuse. It is possible that, unlike people with mental illness, much of the police and legal trouble experienced by people with substance abuse diagnoses is related, not to function or symptoms, but to crimes committed to secure illegal drugs. Consistent with this explanation, the effect of alcohol use disorder is much weaker than that of other forms of substance use disorder (see Table 1). Psychosocial function mediates a greater percentage of the total effect in conditions where psychotic symptoms are common, such as schizophrenia/psychosis, and bipolar 1 disorder. This suggests that future studies of the role of psychotic symptoms should be accompanied by investigations of the role of impaired psychosocial function in the genesis of violence and other behavior leading to police and legal trouble.

These conclusions warrant several caveats. First, in relation to the outcome variable and particularly for disadvantaged groups, engaging in a criminal act is not a necessary prerequisite for being involved with the criminal justice system, for instance, by being apprehended, and people with mental disorders are, in any case, more likely than others to be apprehended when they do commit a criminal offense (Robertson, 1988). Second, NESARC III data derive exclusively from interviews with participants. We were not able to augment the data using collateral sources, for instance, regarding the nature of legal trouble and self-reported psychosocial functioning. Third, while the data used for the mediator variables were derived from reliable instruments, the time period covered by each of those instruments is not the same (past 12-months for AUDADIS, previous 4 weeks for the SF-12v2). This may account for the seemingly greater mediating effect of recent symptoms.

Fourth, the NESARC excluded people who were in prison, who remain more likely than others to suffer from mental disorders (Swanson et al., 2025). This is likely to have lowered rates of police and legal trouble in the sample. Whether it will have altered the role of mediation is less clear, but the possibility cannot be excluded. Fifth, substance abuse is a risk factor for serious police and legal trouble in this sample. Because of the clinical importance of substance abuse as a category of mental disorder, we chose to take this into account by analyzing substance abuse as a predictor separately and not, as we did for other risk factors, as a covariable. While these results confirm a well-established link between substance abuse and legal trouble, a different statistical approach might have yielded different effect sizes.

Finally, data on mediator and outcome variables came from the same 12-month period before the interview. It is possible that for some subjects, an increase in symptoms and a decline in psychosocial functioning were each caused by the subject getting into serious police or legal trouble.

Three considerations make this possibility less likely than would otherwise be the case. First, and while for PTSD prior stress is included in the definition, for neuropsychiatric disorders as a whole, research suggests that stress exerts only a limited effect on symptom prevalence when symptoms are defined, as here, as meeting the threshold for DSM diagnosis (Wang et al., 2024). One recent review suggests an increase in prevalence of <10% (Rao et al., 2022). Second, if these data were a consequence of stress increasing the prevalence of DSM-diagnosable disorder, we would expect role-emotional functioning to exert a larger effect, in terms of percentage of mediation of the total effect, than social functioning (because only role-emotional functioning focuses on the effects of psychological distress [see Method]). Table 2 shows that it exerted less (20.3% vs. 24.5%).

Third, because some symptoms are more likely than others to be provoked by stress, we examined whether the proportion of NESARC subjects with a mental disorder who met criteria for (a) any anxiety disorder and (b) generalized anxiety in the 12 months before interview was higher in subjects who described serious police and legal trouble than in those who did not. The proportion of (a) was slightly lower in subjects with legal trouble (31% vs. 33%) and of (b) slightly higher (16% vs. 14%; Buchanan, Moore, Pittman, & McKee, 2021). While some subjects in legal trouble may have had symptoms that were below the threshold for DSM diagnosis, therefore, and while a fuller understanding requires a study design with multiple, temporarily distinct, data points, it seems unlikely that these results can be explained solely by the stress of police and legal contact causing mental disorder.

One clinical implication of this study, should the results be replicated, concerns the relationship between mental health symptoms in the genesis of patients’ police and legal contacts. Following the failure of high-quality research reliably to link such contacts to particular forms of psychopathology, empirical research and review papers have placed greater emphasis on “criminogenic” correlates of police and legal trouble that include longstanding antisocial traits (Peterson et al., 2014) and early childhood behavior (Swanson et al., 2008). These results suggest that symptoms and signs of mental disorder may nevertheless contribute indirectly to violence and other adverse outcomes and warrant further study.

A second implication concerns the clinical management of risk factors for the various forms of police and legal trouble. The clinical usefulness of risk factors, including younger age, male sex, and a history of violence, has been limited by the inability of otherwise appropriate treatment to change them. The mediators presented here represent not risk factors but components of the mechanisms by which risk factors exert their statistical effects. As such, with improved understanding of their contributions, those mediators may point to therapeutic approaches that can affect the likelihood of adverse legal outcomes. By the same token, the analyses presented here also demonstrate substantial effects that were not mediated by the variables we included. Future research should seek to identify the mediators operating in these cases, also.

## Data Availability

Data were obtained from https://www.niaaa.nih.gov/research/nesarc-iii

## Long descriptions

### Table 1. Long description

The table is structured into three main functional sections: Predictor (Diagnosis), Mediator (Recent psychosocial function and Recent symptoms), and Outcome (Serious police or legal trouble).

* Predictor Columns: Diagnosis, n, and percentage of N.

* Mediator Columns: Social function (mean and S D), Role-emotional function (mean and S D), and Recent symptoms (n and percentage of n).

* Outcome Columns: Serious police or legal trouble (n and percentage of n).

Key data rows include:

* No diagnosis: n = 19,295 (53.14 percent). Social function mean is 52.36. Legal trouble rate is 0.83 percent.

* Any M D (M I, S U, or P D): n = 17,014 (46.86 percent). Social function mean is 47.62. Legal trouble rate is 2.90 percent.

* Schizophrenia/Psychosis: n = 842 (2.33 percent). Social function mean is 42.60. Legal trouble rate is 4.75 percent.

* Any mood disorder: n = 8,293 (22.84 percent). Social function mean is 45.48. Legal trouble rate is 2.89 percent.

* Any anxiety disorder: n = 5,821 (16.03 percent). Social function mean is 44.90. Legal trouble rate is 3.01 percent.

* Any substance use disorder: n = 8,628 (23.76 percent). Social function mean is 47.89. Legal trouble rate is 4.06 percent.

* Any personality disorder: n = 5,746 (15.83 percent). Social function mean is 43.94. Legal trouble rate is 5.48 percent.

* Antisocial: n = 1,600 (4.41 percent). Social function mean is 45.53. Legal trouble rate is 8.25 percent.

Note: S D stands for standard deviation. Higher scores indicate better psychosocial function. Psychiatric categories are not mutually exclusive.

Navigate back to Table 1..

### Table 2. Long description

The table consists of six columns: Mediator, Odds ratio and C I for 12 m serious police or legal trouble, Excess relative risk E R R and C I, E R R due to natural direct effect N D E, E R R due to natural indirect effect N I E, and N I E as percentage of E R R.

Key data points include:

* Any M D (S U, M I, or P D): Odds ratio 2.48. Mediators include Social function (N I E 24.5 percent), Role-emotional function (N I E 20.3 percent), and Symptoms 12 months (N I E 72.6 percent).

* Any M I: Odds ratio 1.78. Mediators include Social function (N I E 42.7 percent), Role-emotional function (N I E 35.7 percent), and Symptoms 12 months (N I E 100 percent).

* Schizophrenia or Psychosis: Odds ratio 2.52. Mediators include Social function (N I E 42.2 percent), Role-emotional function (N I E 40.5 percent), and Symptoms 12 months (N I E 49.4 percent).

* Bipolar 1: Odds ratio 2.01. Mediators include Social function (N I E 53.8 percent), Role-emotional function (N I E 52.4 percent), and Symptoms 12 months (N I E 71.1 percent).

* Any substance use disorder: Odds ratio 2.16. Mediators include Social function (N I E 18.9 percent), Role-emotional function (N I E 14.0 percent), and Symptoms 12 months (N I E 54.0 percent).

All p-values for odds ratios and excess relative risk are less than 0.05. Covariates include age, sex, and history of violence.

Navigate back to Table 2..
